# The m6A/m5C/m1A regulator genes signature reveals the prognosis and is related with immune microenvironment for hepatocellular carcinoma

**DOI:** 10.1186/s12876-023-02776-6

**Published:** 2023-05-11

**Authors:** Ting Liu, Lei Sun, Zhi-zhao Li, Kun Yang, Jia-min Chen, Xiao-yi Han, Li-ming Qi, Xin-gang Zhou, Peng Wang

**Affiliations:** 1 Department of Pathology, Beijing Ditan Hospital, Capital Medical University, No. 8 Jing Shun East Street, Chaoyang District, Beijing, 100015 People’s Republic of China; 2 Department of Cardiovascular, Beijing Ditan Hospital, Capital Medical University, No. 8 Jing Shun East Street, Chaoyang District, Beijing, 100015 People’s Republic of China

**Keywords:** m6A/ m5C/m1A regulatory genes, Hepatocellular carcinoma, Prognostic model, Tumor immune microenvironment

## Abstract

**Background:**

RNA methylation is a crucial in many biological functions, and its aberrant regulation is associated with cancer progression. N6-Methyladenosine (m6A), 5-Methylcytosine (m5C), N1-methyladenosine (m1A) are common modifications of RNA methylation. However, the effect of methylation of m6A/m5C/m1A in hepatocellular carcinoma (HCC) remains unclear.

**Method:**

The transcriptome datasets, clinic information, and mutational data of 48 m6A/m5C/m1A regulator genes were acquired from the TCGA database, and the prognostic hazard model was established by univariate and Least absolute shrinkage and selection operator (Lasso) regression. The multivariate regression was performed to determine whether the risk score was an independent prognostic indicator. Kaplan–Meier survival analysis and ROC curve analysis were used to evaluate the predictive ability of the risk model. Decision curve analysis（DCA）analysis was conducted to estimate the clinical utility of the risk model. We further analyzed the association between risk score and functional enrichment, tumor immune microenvironment, and somatic mutation.

**Result:**

The four-gene (YTHDF1, YBX1, TRMT10C, TRMT61A) risk signature was constructed. The high-risk group had shorter overall survival (OS) than the low-risk group. Univariate and multivariate regression analysis indicated that risk score was an independent prognostic indicator. Risk scores in male group, T3 + T4 group and Stage III + IV group were higher in female group, T1 + T2 group and stage I + II group. The AUC values for 1-, 2-, and 3-year OS in the TCGA dataset were 0.764, 0.693, and 0.689, respectively. DCA analysis showed that the risk score had a higher clinical net benefit in 1- and 2-year OS than other clinical features.The risk score was positively related to some immune cell infiltration and most immune checkpoints.

**Conclusion:**

We developed a novel m6A/m5C/m1A regulator genes' prognostic model, which could be applied as a latent prognostic tool for HCC and might guide the choice of immunotherapies.

## Introduction

Hepatocellular carcinoma (HCC) is the most common primary liver cancer all worldwide, characterized by insidious onset, high incidence, rapid growth, strong invasiveness, and high mortality. Because it lacks effective treatments, it is vital to elucidate the molecular mechanism of HCC for developing new diagnostic methods and defining new targets.

RNA methylation has become an essential form of epigenetic modification and is integral to tumor genesis, progression and prognosis [1–3]. 6-methyladenine (m6A), 5-methylcytosine (m5C) and 1-methyladenine (m1A) are the three most common RNA methylation modifications in eukaryotes. RNA methylation includes writers, erasers, and readers. M6A methylation participates in the progression of glioblastoma, hepatocellular carcinoma, breast cancer, colorectal cancer and other cancers [4–8]. M6A methylase METTL3 has a carcinogenic function in human liver cancer. In the orthotopic liver transplantation model, knockdown of METTL3 can reduce the occurrence of liver cancer and lung metastasis. Loss of METTL3 can down-regulate m6A in vivo and in vitro, and reduce the migration, invasion and epithelial-stromal transformation ability of cancer cells [9, 10]. M5C methyltransferase NSUN2 and m5C binding protein YBX1 are overexpressed in bladder cancer and have poor prognosis, promoting bladder cancer development [11]. M5C methyltransferase NSUN4 and binding protein ALYREF are closely linked to HCC prognosis [12]. M1A demethylases ALKBH3 expression is over-regulated in HCC and has a worse outcome. Down-regulation of ALKBH3 inhibits tumor cell proliferation [13].

Our study is to evaluate predictive ability of m6A/m5C/m1A regulators genes for HCC prognosis. The prognostic risk model of four genes (YTHDF1, YBX1, TRMT10C and TRMT61A) was established by univariate Cox regression, Lasso regression and multivariate Cox regression to predict the prognostic characteristics for HCC patients. First, different data sets were conducted to verify the prediction power for risk model. Then we explored the correlation between the risk model and immune cell infiltration, immune checkpoints and tumor mutations of patients to provide a theoretical study basis for the discovering HCC biomarkers and targets for cancer immunotherapy.

## Materials and methods

### Data collection

The RNA sequence, clinic information, and somatic mutation date were downloded from the TCGA-LIHC database (https://portal.gdc.cancer.gov/) as a training cohort, including 374 HCC patients and 50 normal liver tissues. Similarly, gene expression data and clinical information were also acquired from LIRI-JP in ICGC database (https://dcc.icgc.org/) as a validation cohort, including 232 HCC tissues.

### Screening of differentially expressed genes (DEGs)

Based on previously published literature, we chose 23 m6A regulator genes, 15 m5C regulator genes, and ten m1A regulator genes for study [14–17]. M6A regulator genes included METTL3, METTL14, METTL16, YTHDF1, YTHDF2, YTHDF3, YTHDC1, YTHDC2, RBM15, RBM15B, RBMX, IGF2BP1, IGF2BP2, IGF2BP3, KIAA1429, FMR1, LRPPRC, HNRNPA2B1, HNRNPC, ZC3H13, FTO, ALKBH5 and WTAP. M5C regulator genes contained TRDMT1, NSUN1, NSUN2, NSUN3, NSUN4, NSUN5, NSUN6, NSUN7, DNMT1, DNMT2, DNMT3A, DNMT3B, ALYREF, YBX1 and TET2. M1A regulator genes were comprised of TRMT6, TRMT61A, TRMT61B, TRMT10C, ALKBH1, ALKBH3, YTHDF1, YTHDF2, YTHDF3, and YTHDC1. Wilcoxon test was applied to compare the differential expression of these genes HCC and normal tissues. Then we used the CPTAC from UALCAN to test the protein levels in HCC tissues and normal tissues [18]. In addition, immunohistochemical staining images of HCC and normal liver tissues were obtained from the HPA database.

### Establishment and validation for the prognostic signature of m6A/m5C/m1A regulator genes

To study the prognostic significance of m6A/m5C/m1A regulator genes in HCC, we selected differentially expressed genes (DEGs) in HCC for univariate cox regression to screen prognostic genes using the survival package in R and visualized with a forest plot. Then, the Least Absolute Shrinkage selection operator (LASSO) cox regression analysis was performed by glmnet package in R language for subsequent screening. The prognostic risk signature of four m6A/m5C/m1A regulator genes was constructed. The median risk score acted as the cut-off value. Patients with risk scores above the median were included in the high-risk group, and others in the low-risk group [19]. Kaplan–Meier survival curve was applied to compare the prognosis between the high-risk and low-risk groups for HCC patients by the "survminer" package. We used the timeROC package in R to plot ROC curves further to estimate the predictive accuracy of the risk model. Moreover, we conducted univariate and multivariate cox analyses to decide whether m6A/m5C/m1A regulator genes' prognostic risk model could be independent predictors for HCC patients. Subsequently, the stdca.R package was used to perform decision curve analysis (DCA) analysis to evaluate the clinical utility of the risk model. Meanwhile, we adopted the ICGC database to verify the accuracy of the hazard model.

### Immune cell infiltration and immune checkpoint analysis of prognostic risk model

Single-sample gene set enrichment analysis (ssGSEA) was performed using the "GSVA" package to evaluate the level of infiltration of 24 immune cells in the tumor microenvironment (TME) [20]. CIBERSORT (https://cibersort.stanford.edu/) is used for quantifying the proportion of tumor infiltrating immune cells in TME, which can predict the infiltration of various tumor-related immune cells in tumors and is widely used in disease research based on TME [21]. Additionally, we evaluated the differential expression of immune checkpoints in both risk groups.

### GSEA enrichment analysis

For the gene expression data in TCGA cohort, DESeq2 package was utilized to study the difference in gene expression between high and low-risk groups. We applied the ClusterProfiler package to perform the Gene Ontology (GO) enrichment [22]. GO analysis included three aspects: molecular function (MF), cellular component (CC), and biological process (BP). Among these, molecular functions define molecular processes, cellular components define locations where molecular processes occur, and biological processes define biological programs comprised of regulated factors [23]. The gene set enrichment analysis (GSEA) was conducted using the clusterProfiler package [24]. *P* < 0.05 and FDR < 0.25 were considered significant.

### Statistical analysis

Wilcoxon test was adopted for differential expression analysis. Univariate regression and Least absolute shrinkage and selection operator (Lasso) analysis were employed for model construction, and the Kaplan–Meier method analyzed the prognosis. The multivariate regression was used for identifing independent prognostic markers. Statistical significance was set at a *P*-value < 0.05 [25].

## Result

### Differential expression of m6A/m5C/m1A regulator genes in HCC

**Fig. 1:**
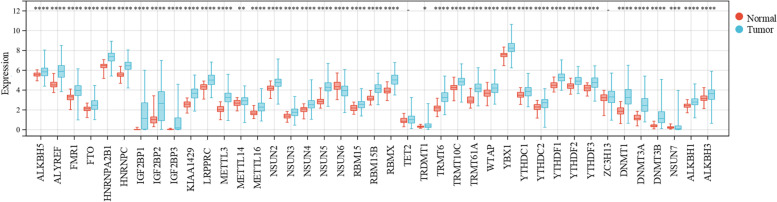
The expression of m6A/m5C/m1A-related genes in HCC patients and normal tissues from TCGA database. (*P < 0.05, **P < 0.01,***P < 0.001)

Because four genes (YTHDC1,YTHDF1-3) in m6A and m1A were duplicated, and there were no expression profile data of NSUN1 and DNMT2 for the TCGA database, we used the Wilcoxon test to analyze the differential expression of 42 m6A/m5C/m1A regulator genes from 374 HCC tissues and 50 normal tissues. The results showed that thirty-nine genes were up-regulated, one gene (NSUN6) was down-regulated, and two genes (ZC3H13, TET2) did not have a significant difference in HCC and normal tissues (Fig. 1).

### Establishment for prognostic risk model of m6A/m5C/m1A regulator genes

**Fig. 2:**
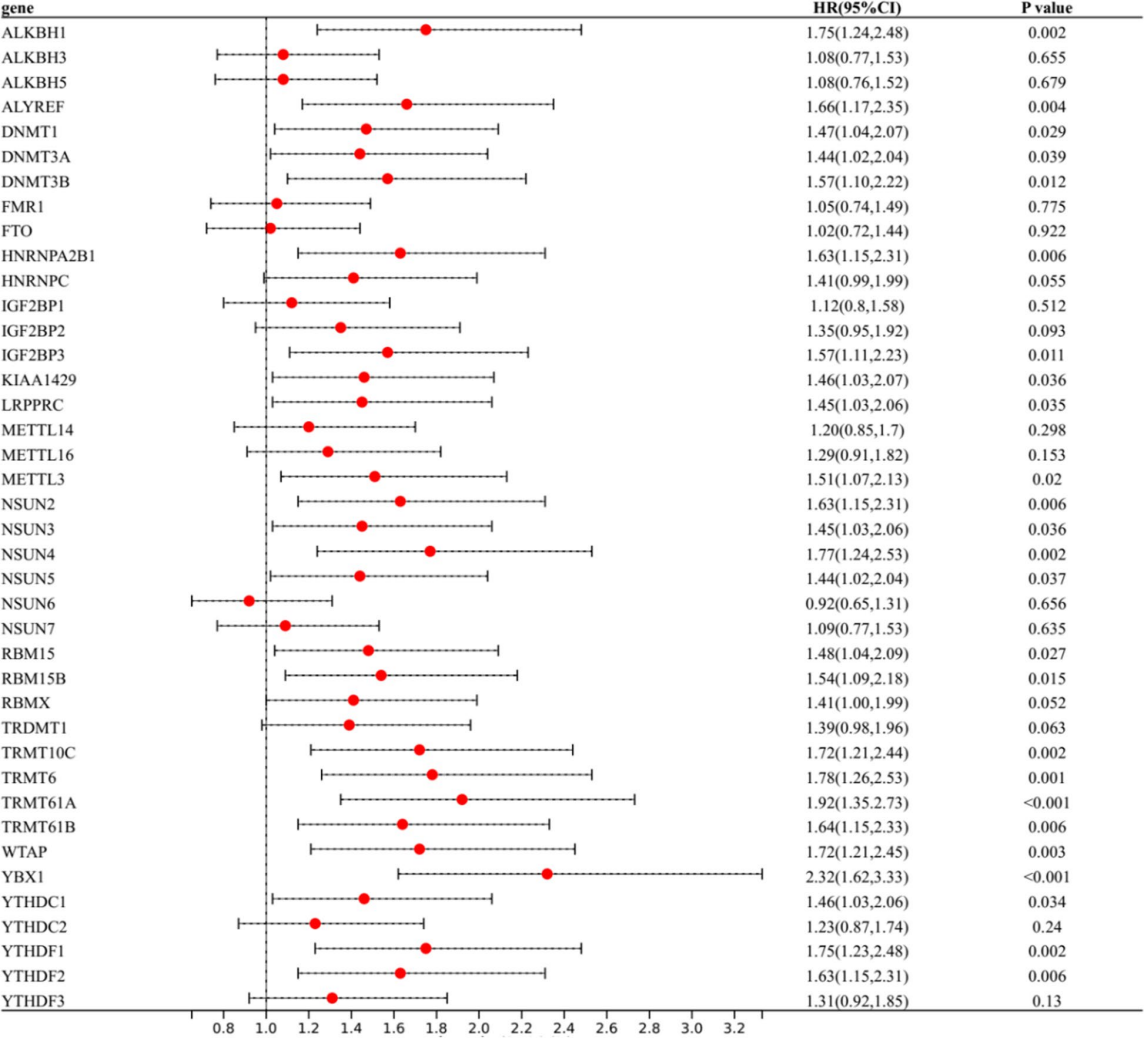
The relationship between differentially expressed genes of m6A,m5C and m1A regulator genes and overall survival (OS) through univariate cox regression

**Fig. 3:**
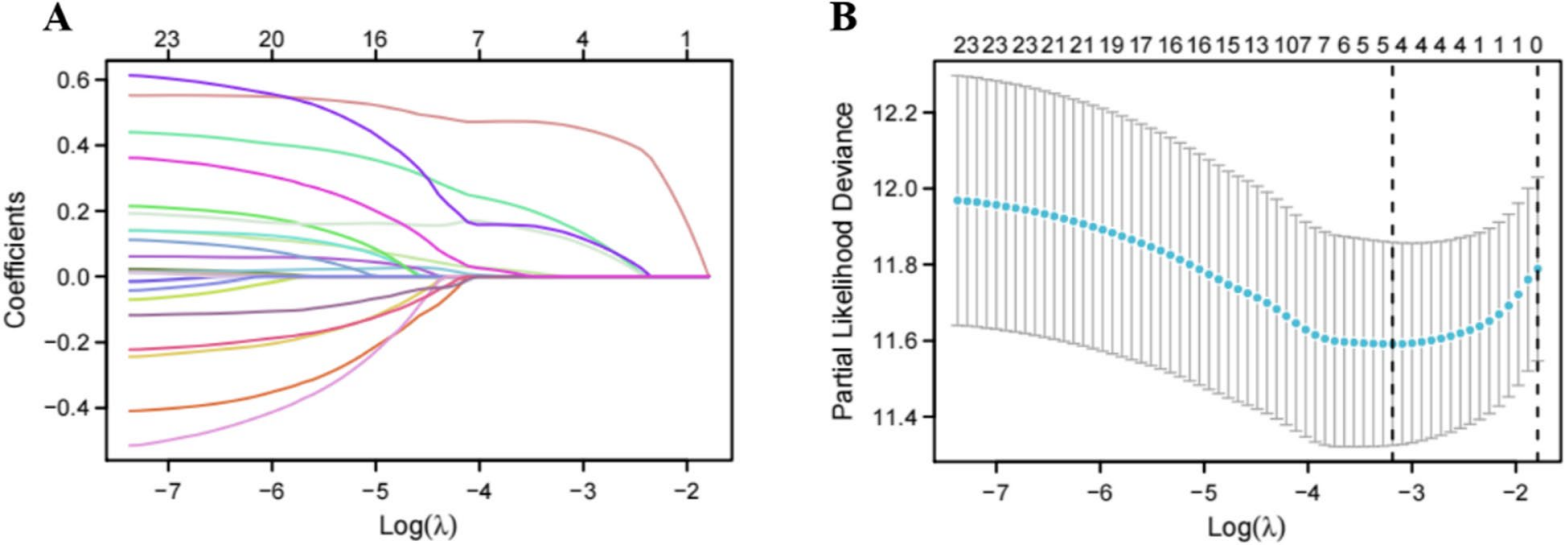
Identification of m6A/m5C/m1A regulaor genes' risk model. **A** Cross validation for tuning parameter selection in the Lasso regression analysis. **B** The four key m6A/m5C/m1A regulator genes were selected by the Lasso regression analysis

By univariate cox regression, 25 DEGs were found to affect prognosis (Fig. 2), including ALKBH1, ALYREF, DNMT1, DNMT3A, DNMT3B, HNRNPA2B1, IGF2BP3, KIAA1429, LRPPRC, METTL3, NSUN2, NSUN3, NSUN4, NSUN5, RBM15, RBM15B, TRMT10C, TRMT6, TRMT61A, TRMT61B, WTAP, YBX1,YTHDC1, YTHDF1 and YTHDF2. The four-gene prognostic risk model was constructed by Lasso regression. Risk Score was defined as follows: Risk Score = 0.1319 × YTHDF1 + 0.468 × YBX1 + 0.1538 × TRMT10C + 0.117 × TRMT61A (Fig. 3A, B).

**Fig. 4:**
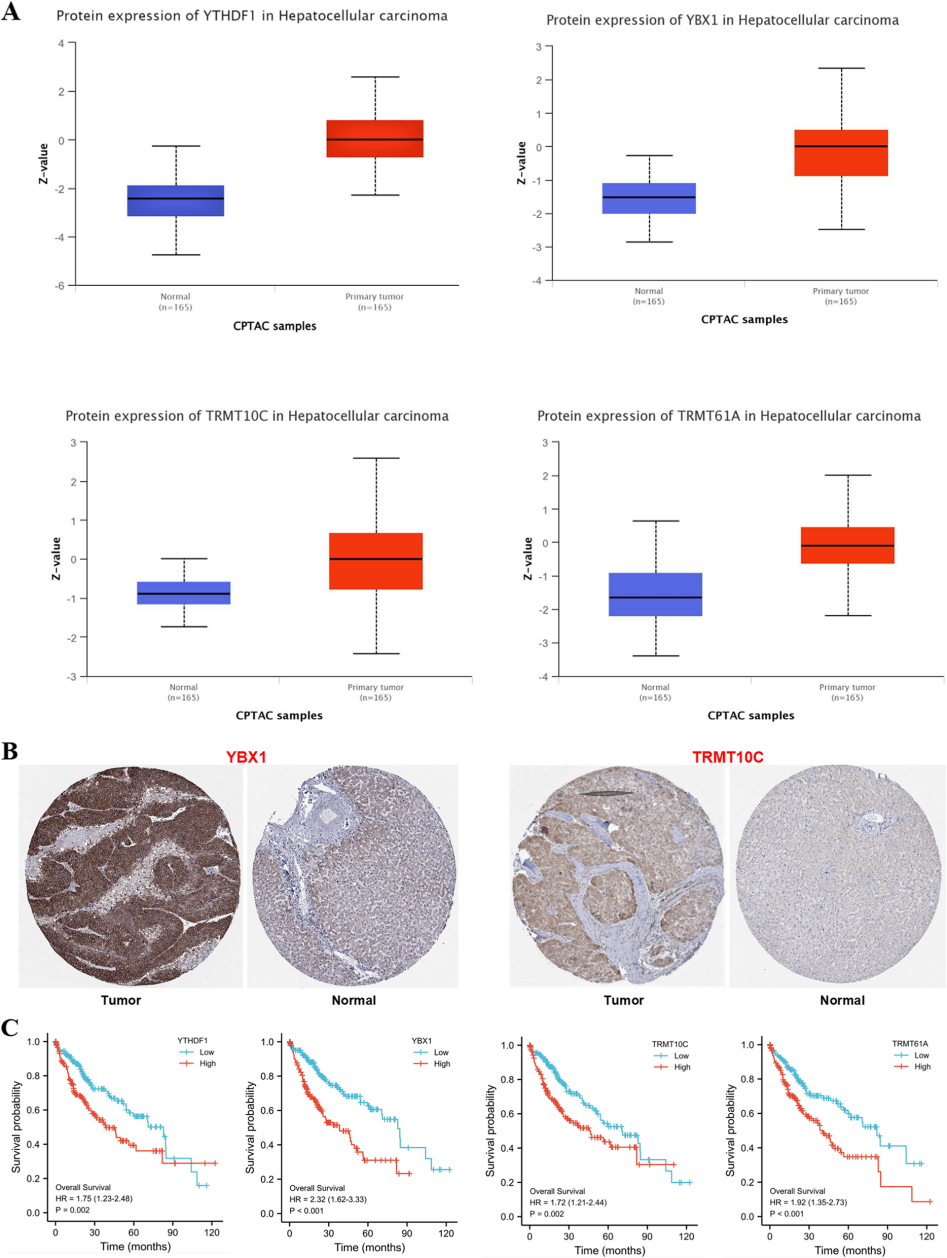
The protein expression and overall survival of four m6A/m5C/m1A regulator genes. **A** The protein expression of four m6A/m5C/m1A regulator genes in CPTAC database. **B** The immunochemistry image of YBX1 and TRMT10C in HCC and normal tissues. **C** The Kalplan-Meier curve of four m6A/m5C/m1A regulator genes’ overall survival

Meanwhile, we studied the potein expression of four genes in the CPTAC from the UALCAN database and immunohistochemical staining from the HPA database. The results demonstrated that the protein expression of four genes (YTHDF1, YBX1, TRMT10C and TRMT61A) was higher in HCC tissues than normal tissues in the CPTAC database (Fig. 4A), and immunochemistry demonstrated that the expression of YBX1 and TRMT10C was higher in HCC than normal tissues (Fig. 4B). Kaplan–Meier survival analysis demonstrated that high expression of four genes (YTHDF1, YBX1, TRMT10C and TRMT61A) were linked with adverse prognosis (*P* < 0.05) (Fig. 4C).

### Development and validation for prognostic risk model

**Fig. 5:**
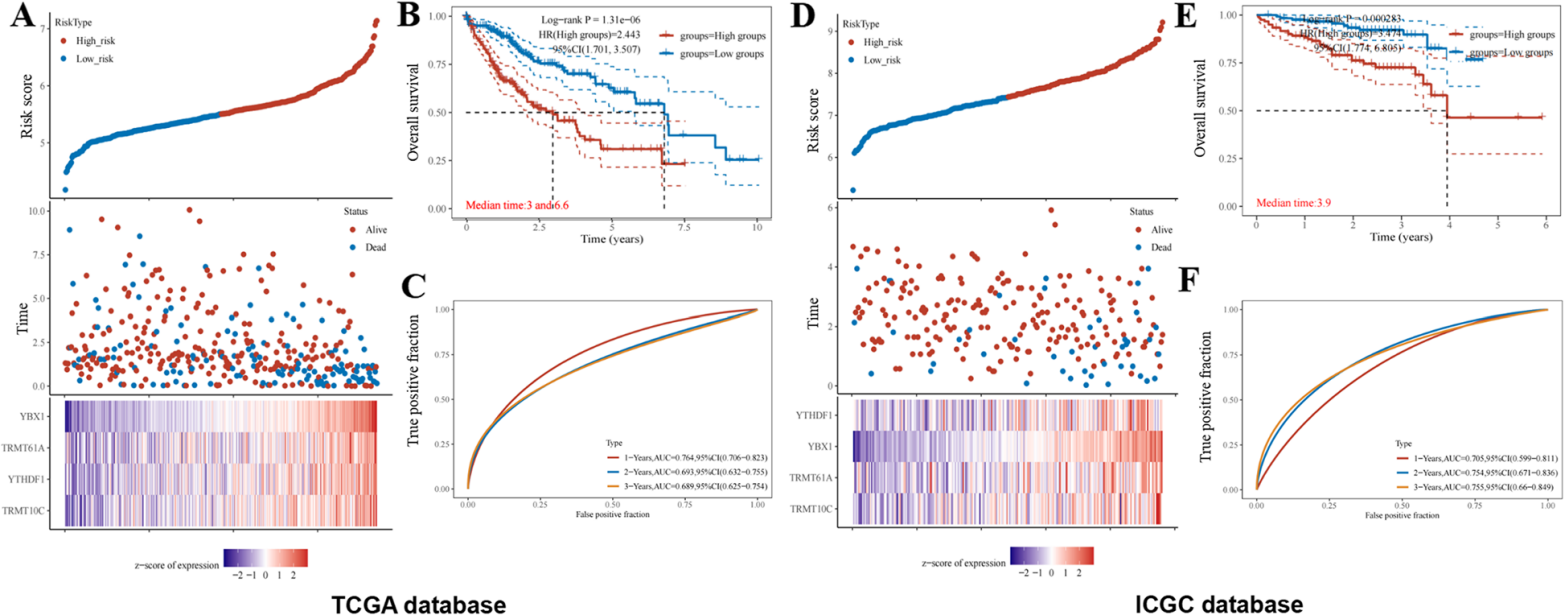
The prognostic model of m6A/m5C/m1A regulator genes in TCGA and ICGC database. **A** The risk score were divided into low risk and high risk group, and the hotmap of four m6A/m5C/m1A regulators expression in TCGA. **B** Kaplan–Meier curves for overall survival in TCGA. **C** ROC curves were used to predict the 1-,2- and 3-years OS of patients in TCGA. **D** The risk score were divided into low risk and high risk group, and the heapmap of four m6A, m5C and m1A-related genes expression in ICGC database. **E** Kaplan–Meier curves for OS in ICGC. **F** ROC curves were used to predict the 1-, 2- and 3-years OS of patients in ICGC

To evaluate and validate the predictive value of the risk score prognostic model, we classified HCC patients into high-risk and low-risk groups based on the median risk score (Fig. 5A). Survival analysis demonstrated that the high-risk group's overall survival rate (OS) was poor (Fig. 5B). In addition, ROC curves displayed that 1-, 2- and 3-year OS were 0.764, 0.693, and 0.689, respectively (Fig. 5C). LIRI-JP in the ICGC database was adopted to confirm the accuracy of the risk model. Similarly, we divided the risk score into high and low-risk groups (Fig. 5D). The high-risk group had poorer OS than the low-risk group (Fig. 5E). AUC of OS in year 1-, 2- and 3- was predicted to be 0.705, 0.754 and 0.755, respectively (Fig. 5F). These findings suggested that the prognostic risk model of m6A/m5C/m1A regulator genes had significant effects on predicting the outcome of HCC.

**Fig. 6:**
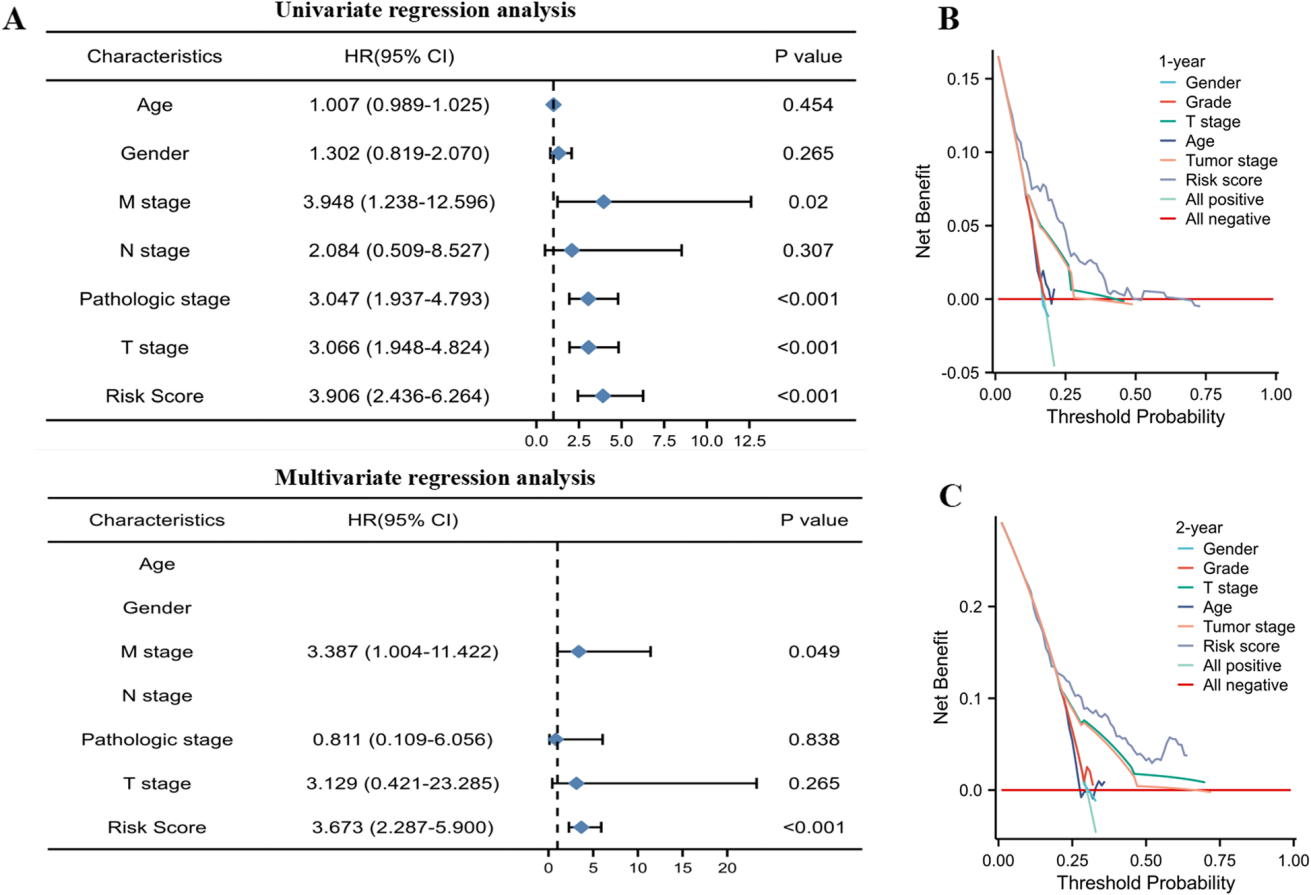
Cox regression analysis and DCA for HCC patients in TCGA database. **A** Univariate and multivariate regression analysis of risk score and clinical features. **B**,**C** The DCA of 1,2-year survival probability for HCC patients in TCGA

To determine whether the risk score was an independent prognostic factor, univariate and multivariate regression analyses were performed. By univariate and multivariate cox regression analysis, we found that risk score and M-stage were independent prognostic factors affecting HCC patients' survival (Fig. 6A). In addition, the DCA showed that compared with other clinical features, the risk score had a higher clinical net benefit in 1-,2-year OS (Fig. 6B, C). These results displayed that the hazard model had good prognostic power for HCC patients.

### Correlation of risk score subgroups with clinical features

**Fig. 7:**
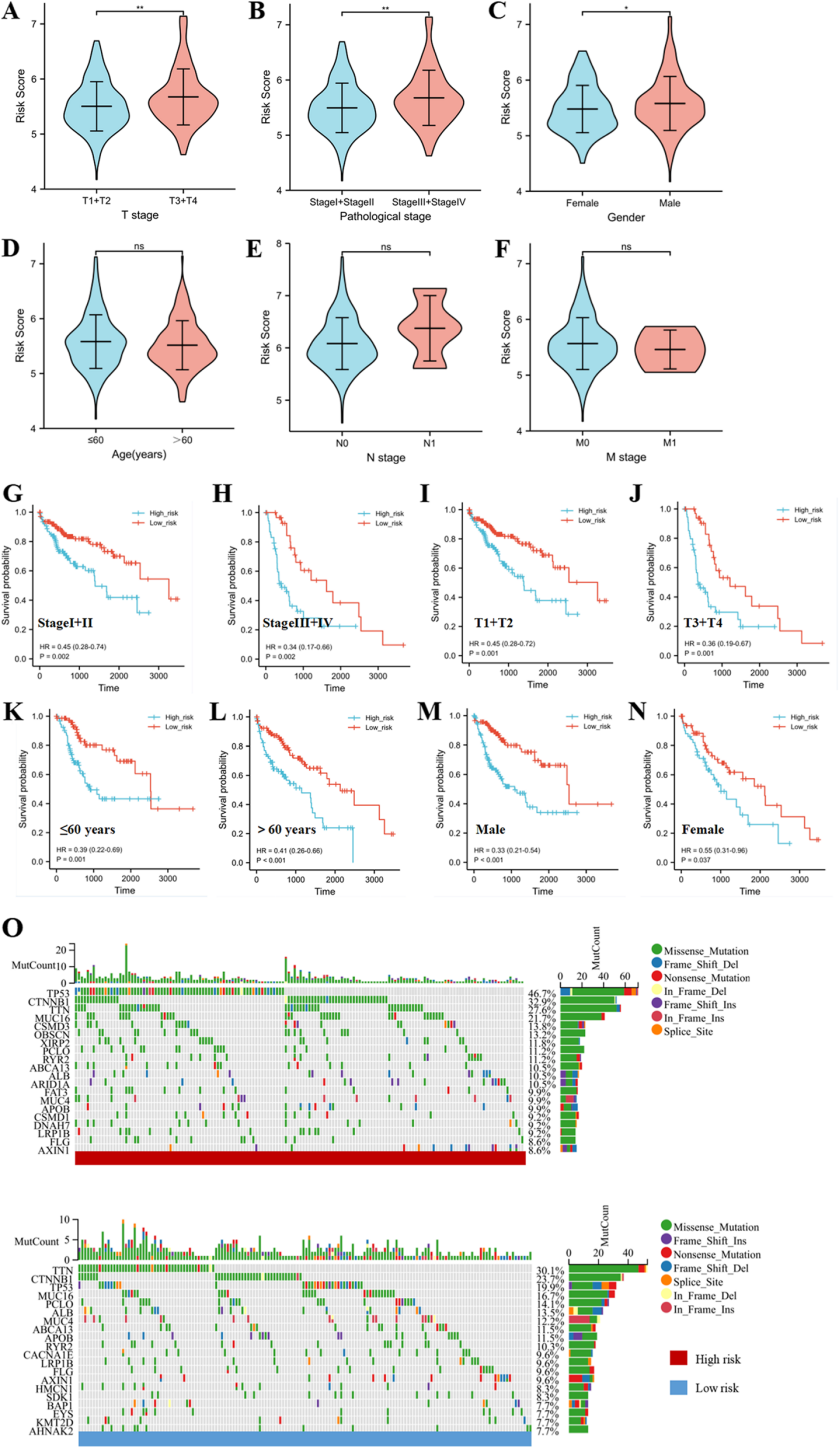
The relationship of risk score with clinical features and somatic mutation. **A-F** The risk score in T stage, pathological stage, gender, age, N stage and M stage. **G-N** Kaplan-Merier curve of low risk group and high risk group in stage I + II group, stageIII + IV group, T1+T2 group, T3+T4 group, ≤ 60 year group, > 60 year group, male group, and female group. **O** The somatic mutation in low risk group and high risk group (***P < 0.001,**P < 0.01,*P < 0.05, ns no significance)

We discussed the association between risk score and clinical features, including gender, age, T stage, M stage, N stage, and pathological stage. The risk score significant correlated with gender, T stage, and pathological stage, but with no relation to age, M stage, and N stage (Fig. 7A-F). Kaplan-Merier survival demonstrated that high-risk group had a worse prognosis for the StageI + II group, Stage III + IV group, T1+T2 group, T3+T4 group, ≤ 60 years old group, > 60 years old group, male group, and female group (Fig. 7G-N). All these indicated that risk score had a reliable ability to predict the prognostic risk model. The landscape of somatic mutations suggested that the top three mutations in both risk groups were TP53, CTNNB1 and TTN. The TP53 mutation rate was the highest in high-risk group (46.7%) and the TTN mutation rate was the highest in low-risk group (30.1%) (Fig. 7O).

### Immune cell infiltration and immune checkpoint analysis for prognostic risk model

**Fig. 8:**
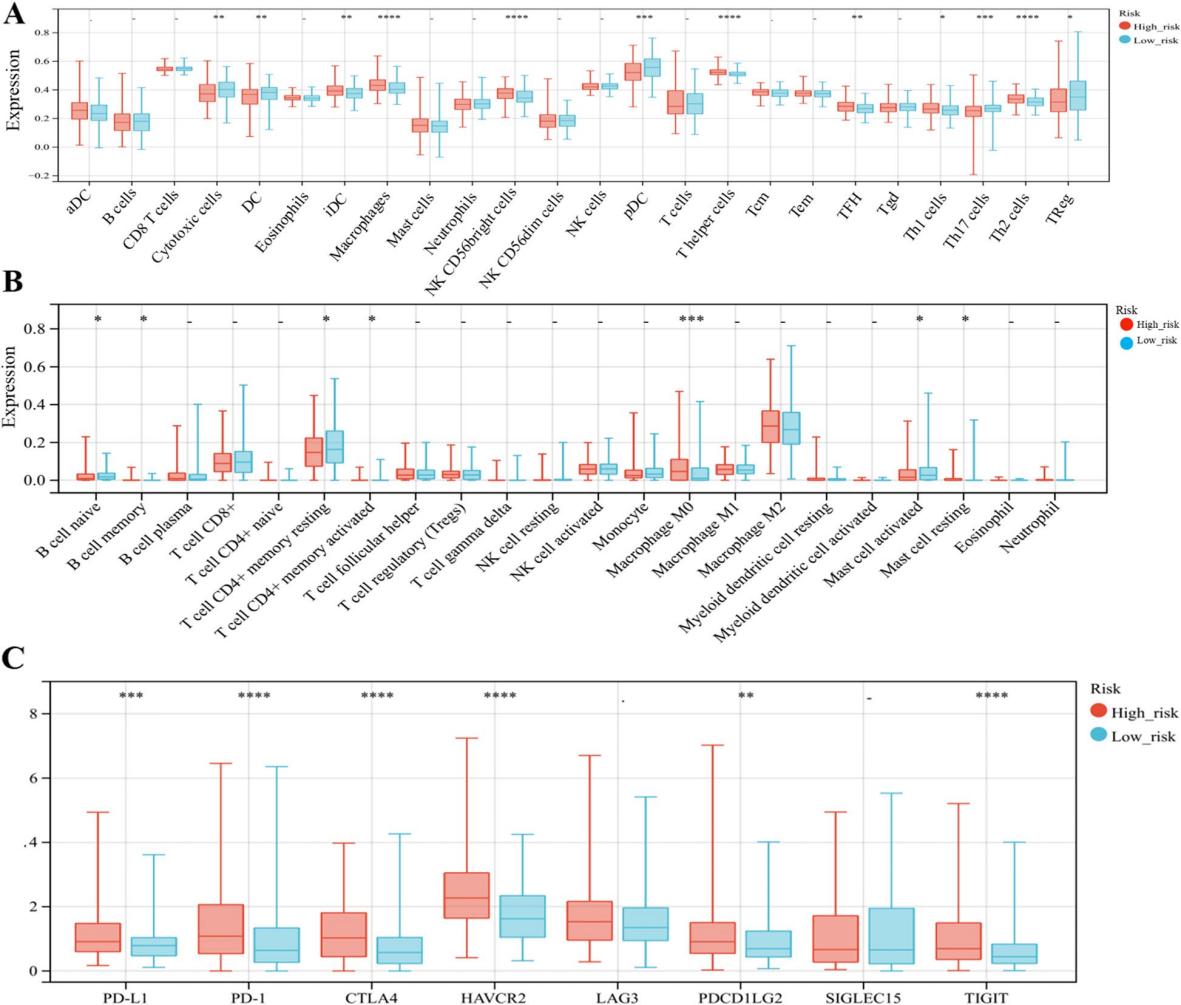
The expression of immune cell infiltration and immune checkpoints in low risk and high risk group. **A** The expression of immune cell infiltration in low risk and high risk group using ssGSEA algorithm. **B** The expression of immune cell infiltration in low risk and high risk group by CIBERSORT algorithm. **C** The expression of immune checkpoints in low risk and high risk group. (***P < 0.001,**P < 0.01,*P < 0.05)

TME is essential in tumor prognosis, malignant progression, and treatment. The composition of immunocyte subsets affects anti-tumor immunity [26]. We used ssGSEA algorithm in the GSVA package to estimate the correlation between risk score and TME for HCC [27]. As these findings showed, the proportion of iDC, macrophages, NK CD56 bright cells, T helper cells, TFH, Th1 cells, and Th2 cells was significantly increased with increasing risk score, and the ratio of cytotoxic cells, DC, pDC, Th17 cells, and Treg decreased substantially with the increase of risk score (Fig. 8A). Then, the CIBERSORT algorithm demonstrated that risk score was positively associated with B cell naive, B cell memory and Macrophage M0, while negatively associated with T cell CD4 + memory resting, T cell CD4 + memory activated, mast cell activated, and mast cell resting (Fig. 8B). Meanwhile, we also investigated the relationship of risk score with immune checkpoints, and discovered that high-risk group had higher expression levels of PD-L1, PD-1, CTLA4, HAVCR2, PDCD1LG2, and TIGIT than low-risk group (Fig. 8C).

### GSEA analysis for prognostic risk model

**Fig. 9:**
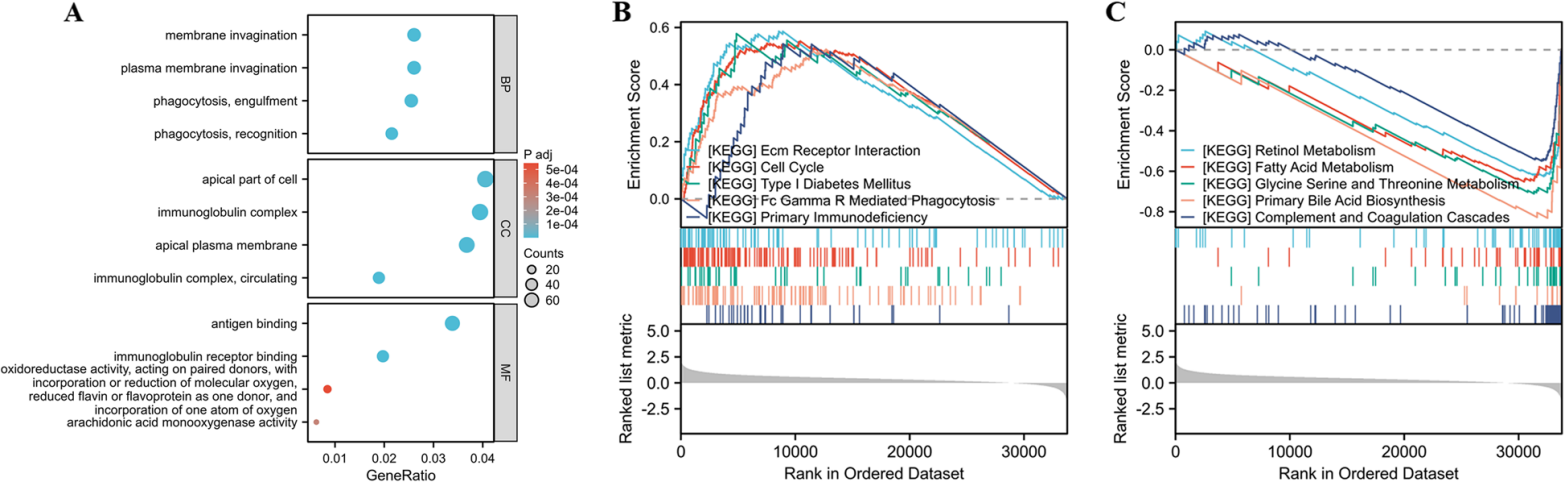
Functional enrichment analysis of risk groups. **A** GO enrichment analysis. **B** The GSEA enrichment in high risk group. **C** The GSEA enrichment in low risk group

To further study the biological process, we employed GO analysis to analyze the gene enrichment analysis of HCC patients in the high and low risk group. Biological process participated in membrane invagination, plasma membrane invagination, et al. The cellular component mainly focused on the apical part of cell, immunoglobulin complex, et al. The molecular function involved antigen binding, immunoglobulin receptor binding, et al. (Fig. 9A).

GSEA enrichment analysis demonstrated that the high risk groups were enriched in ECM receptor interaction, cell cycle, type I diabetes mellitus, FC gamma R mediated phagocytosis, and primary immunodeficiency (Fig. 9B). Conversely, the low-risk groups were significantly involved in retinol metabolism, fatty acid metabolism, glycine serine and threonine metabolism, primary bile acid biosynthesis, and complement and coagulation cascades (Fig. 9C).

## Discussion

Hepatocellular carcinoma (HCC) is a most typical malignant tumors, with more than 740,000 new HCC patients and about 700,000 deaths due to HCC in the world every year [28]. Therefore, finding independent prognostic factors as new targets for advanced treatment of HCC patients and improving patients' survival is cricial. With the rapid development of high-throughput sequencing technology, epigenetic modification, especially RNA methylation modification (mainly including m6A, m5C and m1A), has been paid more attention and developed rapidly over recent years. Many studies have confirmed that RNA methylation is crucial in malignant tumors progression [29–32]. Recent studies have established a prognostic model of skin melanoma base on m6A/m5C/m1A regulator genes [33]. However, only some prognostic models for m6A/m1A/m5C regulator genes have been studied in HCC.

In our study, 48 m6A/m5C/m1A regulator genes were selected for research and then screened out 40 differentially expressed genes for HCC. A four-gene prognostic risk model (YTHDF1, YBX1, TRMT10C and TRMT61A) was constructed using univariate and Lasso regression. The risk score was researched by univariate and multivariate regression, and regarded the risk score as an independent adverse prognostic factors for HCC. Kaplan–Meier analysis was conducted on the high-and low-risk group, and found that the prognosis of the low-risk group was significantly better than that of high-risk group. Moreover, the risk score was related to gender, T stage and pathological stage. We also performed ROC analysis to test the sensitivity and specificity of the hazard model, and calculated the corresponding AUC values at 1-, 2- and 3- years, respectively. The DCA analysis considered the risk score a higher clinical net benefit than the clinical characteristics. The research results showed that the risk model had good prediction ability. The validation set ICGC also demonstrated that the prognostic risk model had good predictive value.

As readers of m6A, YTHDF1 has high expression in colon cancer, and knockdown of YTDHF1 expression significantly inhibits the CRC cells tumorigenicity in vitro, mouse xenograft tumors the growth and Wnt/β-catenin pathway activity in CRC cells [34]. YTHDF1 is strongly expressed in HCC and leads to a bad prognosis. Experiments in vitro and in vivo have demonstrated that YTHDF1 could promote liver cancer's proliferation and metastasis [35, 36]. YTHDF1 playes a vital role in the epithelial-mesenchymal transformation of HCC. After YTHDF1 is knocked out, EMT markers N-cadherin and vimentin expression are suppressed, and E-cadherin is up-regulated. YTHDF1 may activate AKT/GSK-3 β/β- Catenin signaling pathway and promotion of EMT to enhance HCC cells' proliferation, invasion, and metastasis [37]. YBX1, a newly discovered m5C binding protein that regulates the stability of mRNA in cytoplasm, and is overexpressed in most cancers, is associated with tumor cell proliferation, anti-apoptosis, migration and prognosis, and is expected to be an ideal diagnostic biomarker and a candidate therapeutic target [38]. YBX1's high expression in breast cancer is associated with low survival, drug resistance, and high recurrence rates for all subtypes, indicating the potential importance of YBX1 as an oncogene in breast cancer [39]. It is reported that YBX1 directly binds to lncRNALINC00312 to promote lung cancer cell invasion, migration, and angiogenesis [40]. TRMT10C is overexpressed in ovarian and cervical cancer, and has a poor prognosis, which may promote tumorigenesis by affecting C-Myc-related pathways [41]. TRMT61A and TRMT6 forme a complex of m1A methyltransferase, which is up-regulated in HCC and is linked with poor survival. TRMT6/TRMT61A enhances m1A methylation in tRNA subsets to increase PPARδ translation, which triggers cholesterol synthesis, activates Hedgehog signaling, and ultimately promotes self-renewal and tumorigenesis of liver CSC [42]. Our study also confirmed that the protein expression of four genes was higher in HCC than corresponding normal tissues, which was consistent with the above research.

We performed enrichment and immunological analysis to explore the biological processes involved in HCC. GSEA enrichment analysis showed that the high-risk group was engaged in ECM receptor interaction, cell cycle, type I diabetes mellitus, FC gamma R mediated phagocytosis and primary immunodeficiency. Studies have shown that dysregulation of these processes will lead to tumorigenesis and progression [43–47]. TME contains fibroblasts, vascular endothelial cells, immune cells and cytokines released by cells, which are closely linked with tumor proliferation, invasion, and metastasis. More and more studies have shown that RNA methylation relates to TME [48, 49]. Zhao et al. construct a prognostic model of m6A, which reveals the TME features of HCC patients with distinct m6A expression patterns and find that the high LRPPRC m6A modulator expression had depletion of T cells, cytotoxic cells, dendritic cells, and cytolytic activity response [50]. Yong Liu et al. divide m5C methylation regulators into three different m5C clusters, and the results show that m5C modification patterns play a crucial role in the TME for HCC [51]. It has been reported that m1A-score model in HCC is correlated with TME [52]. Based on the above research, we studied the correlation the risk model for m6A/m5C/m1A regulator genes with TME. We used the ssGSEA algorithm to analyze the association of risk score with immune cell infiltration. The findings suggested that the risk score was positively correlated with iDC, macrophage, NK CD56 bright cells, T helper cells, TFH, Th1 cells, and Th2 cells. Macrophages, as the first line of defense for immune defense, play an essential part in all stages of tumor genesis and development, and are central regulators of TME [53]. It also promotes tumor angiogenesis and tumor metastasis [54–57]. Th2 expression level increased with increasing risk scores, possibly due to Th1/Th1 drift caused by Th1/Th2 imbalance and tumor cells escaping from immune surveillance, leading to tumor occurrence. CIBERSORT algorithm also indicated that the high-risk group had higher infiltration of B cell naive, B cell memory and Macrophage M0 and lower infiltration of T cell CD4 + memory resting, T cell CD4 + memory activated, mast cell activated, and mast cell resting.

With the discovery of tumor biology and immunology, tumor immunotherapy has become a new way of tumor therapy. The discovery of immune checkpoints provides a new idea for tumor immunotherapy. Immune checkpoints are negative immunomodulatory molecules expressed on the surface of immune and tumor cells, and are closely related to tumor proliferation, invasion, metastasis and prognosis assessment.They are a good target for tumor therapy. Our study chose eight common immune checkpoints, containing PD-L1, PD-1, CTLA4, HAVCR2, PDCD1LG2, TIGIT, LAG3 and SIGLEC15, and discovered increased expression of PD-L1, PD-1, CTLA4, HAVCR2, PDCD1LG2, and TIGIT in high-risk group. The upregulation of immune checkpoints in TME benefits tumor growth [58]. High-risk patients might benefit from immunotherapy.

In summary, we constructed a risk model of m6A/m5C/m1A regulator genes in HCC and discovered that these genes had particular clinical value in diagnosis and prognosis. However, this study also had some limitations. We perfomed bioinformatics analysis to study the relationship between m6A/m5C/m1A regulator genes and prognosis for HCC patients. Its mechanism in HCC remained unclear. Many experiments are needed to investigate further the role of m6A/m5C/m1A regulator genes in HCC.

## Conclusion

We established a prognostic signature of four-gene for predicting HCC prognosis and were a potential predictor in patients with HCC. Our study could perform individual-based treatment and might help to improve the prognosis of HCC patients.

## Data Availability

The results shown here are in whole or part based upon data generated by TCGA(https://portal.gdc.cancer.gov/), ICGC (https://dcc.icgc.org/), CIBERSORT (https://cibersort.stanford.edu/) databases.
